# Novel neuronal surface autoantibodies in plasma of patients with depression and anxiety

**DOI:** 10.1038/s41398-020-01083-y

**Published:** 2020-11-23

**Authors:** Shenghua Zong, Carolin Correia-Hoffmann, Marina Mané-Damas, Nils Kappelmann, Peter C. Molenaar, Gerard van Grootheest, Brenda W. J. H. Penninx, Rob P. W. Rouhl, Mario Losen, Pilar Martinez-Martinez

**Affiliations:** 1 Department of Psychiatry and Neuropsychology, School for Mental Health and Neuroscience, Maastricht University, Maastricht, the Netherlands; 2 Department of Psychiatry and Neuroscience Amsterdam, Amsterdam UMC, Vrije Universiteit, and GGZ inGeest, Amsterdam, the Netherlands; 3 Department of Neurology, Maastricht University Medical Center (MUMC+), Maastricht, the Netherlands; 4 School for Mental Health and Neuroscience, Maastricht University, Maastricht, the Netherlands; 5 Academic Centre for Epileptology Kempenhaeghe/MUMC+, Maastricht, the Netherlands; 6 Present Address: Centre for Biomedical Research, University of Algarve Gambelas Campus, Faro, Portugal; 7 Present Address: Department of Translational Research in Psychiatry, Max Planck Institute of Psychiatry, Munich, Germany; 8 Present Address: International Max Planck Research School for Translational Psychiatry (IMPRS-TP), Munich, Germany

**Keywords:** Depression, Molecular neuroscience, Psychiatric disorders

## Abstract

Neuronal surface autoantibodies (NSAbs) against various antigens cause autoimmune encephalitis. Some of these antigens are also involved in the pathology of depression and anxiety. To study whether NSAbs are more common in plasma of individuals with depression and anxiety than in controls, and to investigate if NSAbs correlate with disease status, plasma samples of 819 individuals with a current diagnosis of depression and/or anxiety, 920 in remission and 492 individuals without these disorders were included in this study. Samples were tested by a combination of immunohistochemistry (IHC), staining on live rat hippocampus neurons and cell-based assay (CBA). By IHC, 50 (2.2%) samples showed immunoreactivity to rat brain tissue, with no significant differences between the aforementioned groups (22/819 vs 18/920 vs 11/492, *P* > 0.99). In addition, eight IHC positive samples were positive for NSAbs on live neurons (7/819 vs 0/920 vs 1/492, *P* = 0.006). The IHC-staining patterns of these eight samples were atypical for autoimmune encephalitis and accordingly, they tested negative for known NSAbs by CBA. No obvious difference in the clinical characteristics between individuals with or without NSAbs was observed. In conclusion, novel NSAbs were rare but predominately found in patients with current anxiety or depression indicating they might affect mental health in a small group of patients.

## Introduction

Depression and anxiety disorders are among the most common illnesses and their economic burden ranks among the top-five of all diseases^1,2^. Although these disorders may share a similar cluster of symptoms, they are believed to be caused by heterogeneous pathophysiologic mechanisms. Immune dysregulation has been observed in patients with depression and anxiety^3–6^ and a high prevalence of these disorders was found in autoimmune diseases^7–10^, which may suggest a link between autoimmunity and depression or anxiety.

It has been reported that certain neuronal surface autoantibodies (NSAbs) are associated with isolated symptoms of psychosis^11^. These autoantibodies are pathogenic by degrading or blocking neurotransmitter receptors, ion channels, or associated proteins, ultimately leading to dysfunction of neural signal transduction in most cases^12^. Neurotransmitter transporters and receptors are clearly implicated in the pathology of depression and anxiety and they are targeted therapeutically with anti-depressant drugs^13,14^. Thus, there is a good reason to consider the possibility that certain NSAbs may constitute one of the putative causes of depression and anxiety^15–17^.

Studies investigating the prevalence of NSAbs in psychiatric diseases have mainly focused on autoantibodies against the *N*-methyl-d-aspartate receptor (NMDAR) in psychotic disorders but the possible role of neuronal autoantibodies in depression and anxiety has received little attention thus far^18–23^. A few studies have included depression and anxiety cohorts but have not revealed any instances of specific neuronal autoantibodies related to these disorders^24,25^. Limited cohort size and the lack of further validation of the results, led to inconclusive evidence.

Autoimmune encephalitis caused by NSAb is commonly diagnosed by cell-based assays (CBA), including fixed and live CBA, which are designed to detect autoantibodies to known, well-defined antigens. When extending neuronal autoantibody detection from neurological disorders to new disorders (as in this case depression or anxiety), using methods that can also detect novel neuronal antibodies would be preferable. Therefore, immunohistochemistry (IHC) using rat brain tissue, optimized for the preservation of cell-surface antigens, or cultured rat neurons, are promising techniques^26^, albeit with the limitation that only cross-reactive NSAbs with human antigens would be detected by these methods.

In this study, we investigated autoantibodies binding to brain tissue, with a focus on NSAbs in the plasma from a large cohort of patients with depression and/or anxiety as well as from control individuals. We followed a tandem procedure, combining different methods including IHC on rat brain, staining on live-cultured rat hippocampal neurons and CBA as reported^27^.

## Material and methods

### Participants and samples

The Netherlands Study of Depression and Anxiety (NESDA) is an ongoing longitudinal cohort study designed to investigate the course of depression and anxiety disorders for several years. The inclusion and exclusion criteria were described^28^. Participants were recruited from primary care practices and specialized mental health institutions in the regions of Amsterdam, Leiden and Groningen, Drenthe and Friesland of the Netherlands. In brief, 2981 participants were included at baseline (Wave 1), 2596 were available at the 2-year follow-up (Wave 3) and 2256 at the 6-year follow-up (Wave 5).

The current study initially used all available plasma samples collected by NESDA at Wave 3 and the corresponding clinical information. The study included 819 individuals with a current diagnosis of depression and/or anxiety, 920 in remission and 492 individuals without these disorders. If individuals tested positive by staining on live neurons at Wave 3, we then also retrieved their clinical information and plasma samples from Wave 1 and Wave 5. The ethical committee of participating universities approved the research protocol and all respondents provided written informed consent. The Composite Interview Diagnostic Instrument—lifetime version 2.1—was used to diagnose depression and anxiety disorders according to the Diagnostic and Statistical Manual of Mental Disorders—Fourth Edition algorithms. The whole cohort consisted of individuals with depression and/ or anxiety disorders, current (depression and /or anxiety present in the 6 months prior to assessment) or in remission (depression and/or anxiety diagnosed earlier in life but not present in the 6 months prior to assessment), and controls without these disorders. Details are given in Table 1. Plasma samples were stored at −80 °C. Working aliquoted samples were kept at −20 °C.

**Table 1:** The demographic characteristics of the NESDA cohort (at the 2-year follow-up).

Clinical values	Current depression/anxiety^a^ (*n* = 819)	Remitted depression/anxiety^b^ (*n* = 920)	Control group (*n* = 492)	*P* value^c^
Mean age (SD)	44.9 (12.3)	44.5 (13.2)	43.6 (14.6)	0.22
Age range (y)	19–66	19–68	20–66	
Female (%)	552 (67.4%)	606 (65.9%)	291 (59.1%)	0.008
*Mental disorder subgroups*				
Depression	520 (63.5%)	758 (82.4%)	–	
Major depressive disorder	475 (91.3%)	745 (98.2%)	–	
Dysthymia	198 (38.1%)	210 (27.7%)	–	
Anxiety	608 (74.2%)	612 (66.5%)	–	
Panic disorder with agoraphobia	107 (17.6%)	130 (21.2%)	–	
Panic disorder without agoraphobia	140 (23.0%)	149 (24.3%)	–	
Social phobia	306 (50.3%)	266 (43.4%)	–	
Generalized anxiety disorder	166 (27.3%)	259 (42.3%)	–	
Agoraphobia without panic disorder	125 (20.6%)	109 (17.8%)	–	
Using psychiatric medication in recent 2 years^d^	324 (39.6%)	195 (21.1%)	23 (4.7%)	
*Somatic diseases*^*d*^				
Diabetes	52 (6.3%)	45 (4.9%)	17 (3.5%)	0.066
Stroke	12 (1.6%)	17 (1.8%)	12 (2.4%)	0.42
Arthritis or arthrosis	193 (23.6%)	160 (17.4%)	86 (17.4%)	0.002
Chronic none specific lung disease	118 (14.4%)	110 (12.0%)	45 (9.1%)	0.019
Rheumatism (fibromyalgia, SLE, rheumatoid arthritis)	87 (10.6%)	53 (5.8%)	14 (2.8%)	<0.001
Tumor	50 (6.1%)	64 (7.0%)	27 (5.5%)	0.52
Ulcer	16 (2.0%)	10 (1.1%)	2 (0.4%)	0.047
Intestinal disorders	166 (20.3%)	134 (14.6%)	35 (7.1%)	<0.001
Allergies (hay fever, eczema)	267 (32.6%)	312 (33.9%)	140 (28.5%)	0.22
Thyroid disease (Graves disease, hyperthyroidism)	35 (4.3%)	35 (3.8%)	17 (3.5%)	0.76
Head injury	20 (2.4%)	22 (2.4%)	5 (1.0%)	0.15
*Sickness 1 week prior to blood drawn*^*d*^				
Fever	38 (4.6%)	42 (4.6%)	18 (3.7%)	0.69
Cold	233 (28.4%)	252 (27.4%)	135 (27.4%)	0.87

^a^Depression and/or anxiety present in the 6 months prior to assessment.

^b^Lifetime depression and/or anxiety diagnosis, but not in the 6 months prior to assessment.

^c^*t* test is used for comparing the age difference between groups and the chi-square test is used for comparison of gender, somatic diseases, and sickness prior to blood drawn between different groups.

^d^The number is dependent on questionnaires when the answer is “yes”.

### Serological analyses

A scheme of the workflow is shown in Supplementary Fig. 1.

### *Immunohistochemistry*

Fresh adult rat brains (Lewis, male, 12 weeks) were fixed in 4% paraformaldehyde for 1 h at 4 °C, dehydrated in 30% sucrose for 48 h at 4 °C, frozen in liquid nitrogen and cut into 5–7 µm thick sagittal serial sections. These sections were incubated at room temperature with 0.3% H_2_O_2_ for 15 minutes and then with 5% goat serum for 1 h. Subsequently, they were incubated with 200 µl plasma from the NESDA cohort (1:200 diluted in 5% goat serum) overnight at 4 °C and subsequently with biotinylated goat anti-human IgG Fcγ (1:3200 in 5% goat serum, Jackson Immunoreserach, #109-066-008) for 2 h at room temperature. Each step was followed by three washing steps with phosphate-buffered saline. The reactivity was visualized using avidin-biotin-peroxidase (Vector laboratory, Inc., # PK 6100) and 3,3-diaminobenzidine (Sigma, #868272-85-9). After dehydration, slides were mounted using dibutyl phthalate in xylene (Klinipath, #C933401) or Entellan (Millipore Sigma, #1.07961.0100). Each staining included a positive control serum from patients with autoimmune encephalitis and autoantibodies to NMDAR, α-amino-3 hydroxy-5-methyl-4-isoxazolepropionic acid receptor (AMPAR) or dipeptidyl-peptide-like protein 6 (DPPX), and a negative control from a healthy individual. Images of rat brain IHC were taken by the iScan HT slide scanner (Ventana; ×20 objective) and visually graded 0–3 (Ventana Image Viewer) for the hippocampal immunoreactivity of plasma IgG, based on the intensity and contrast of the staining. All samples were randomized and coded before the autoantibody test. Investigators were blinded to the code until the scoring was completed. IHC was also used to define the detected autoantibody titers. For details, see Supplementary Methods.

### *Cell-based assays*

#### *Fixed CBA*

To test autoantibodies to known neuronal antigens, including NMDAR, AMPAR, γ-aminobutyric acid receptor subunits A and B (GABAAR, GABABR), leucine-rich glioma-inactivated 1 (LGI1), contactin-associated protein-like 2 (CASPR2), glutamic acid decarboxylase 65 and 67 (GAD65 and GAD67), HEK cells (checked routinely for mycoplasma contamination) were transfected with plasmids carrying the recombinant cDNA encoding these proteins. The source of the plasmids is described in supplementary methods. HEK293 cells were plated on coverslips and transfected (see supplementary methods). Cells were fixed in 3.6% formaldehyde (TAAB, #F006) for 10 minutes and permeabilized with 0.3% Triton-X-100 for 10 minutes. After blocking with 1% bovine serum albumin (BSA) for 1 h, cells were co-incubated with human sera (1:40 diluted in 1% BSA) and a commercially available antibody targeting a specific antigen for 1 h at room temperature. A complete overview of the antibodies used can be found in supplementary Table 1. Human IgG was visualized using goat anti-human IgG Fcγ Alexa488 and cell nuclei were stained with 4’, 6-diamidino-2-phenylindole in the mounting medium. See supplementary methods for details regarding the primary and secondary antibodies used for the detection of the neuronal antigens as well as for additional methodological information for this section.

Live cell-based assay was performed as previously described for fixed CBA with the difference that antigens were expressed with fluorescent reporter proteins if available and human serum was incubated with living cells instead of fixed and permeabilized cells. Transfected HEK cells were incubated with human serum (1:50 diluted in DMEM with 1% BSA and 25 mM 4-(2-hydroxyethyl)-1-piperazineethanesulfonic acid at room temperature for 1 h. Cells were then fixed with 3.6% formaldehyde and incubated with the corresponding secondary antibodies (Supplementary Table 1), mounted and analyzed as described above.

#### *Pre-absorption test by live CBA and IHC*

To validate if an IHC pattern was given by autoantibodies detected by live CBA in each of the CASPR2-positive samples, a pre-absorption test was performed as described^29^. For absorption of CASPR2 antibodies, 500 µl of CASPR2 antibody-positive plasma or other plasma samples as controls (diluted 1 in 200) were added to the CASPR2 transfected HEK cells growing in 24 well plates and incubated for 1 h at 37 °C. Then the supernatant was collected and applied to the next well of the CASPR2 transfected HEK cells. These procedures were repeated four times. Afterwards, the supernatant was collected to perform IHC staining on rat brain slice as explained above.

#### *Staining on live neurons*

To test if autoantibodies directly target neuronal surface proteins, rat hippocampal neuronal staining was performed as described previously^27,30^, with small modifications. In brief, neurons were incubated with patients’ sera, followed by incubation with anti-human IgG fluorescent-labeled secondary antibody (supplementary Table 1) for 1 h and mounted with mounting medium containing DAPI; all the steps were performed at room temperature. A positive control with autoantibodies to NMDAR from an encephalitis patient and negative controls from healthy individuals were included. The results were scored as negative, weak positive or strong positive according to the fluorescent signal on the surface of neurons. Neuronal staining in positive samples was confirmed using an anti-microtubule-associated protein 2 (MAP2, Millipore, AB5622, 1:1500 diluted in 1% BSA, after a 10 min permeabilization step), followed a by goat anti-rabbit IgG Alexa 594 staining.

### Statistical analysis

Fisher’s exact test was used to compare the prevalence of positive IHC samples between groups (disorders and controls, or current disorder, remission and controls). Chi-Square test was performed for categorical values (sex) and ANOVA for continuous values (age). All analyses were performed using IBM SPSS Statistics version 23.0. The differences were considered statistically significant when *P* values were below 0.05.

## Results

### IHC studies: 2.2% of the samples were found positive with 11 novel patterns

**Fig. 1:**
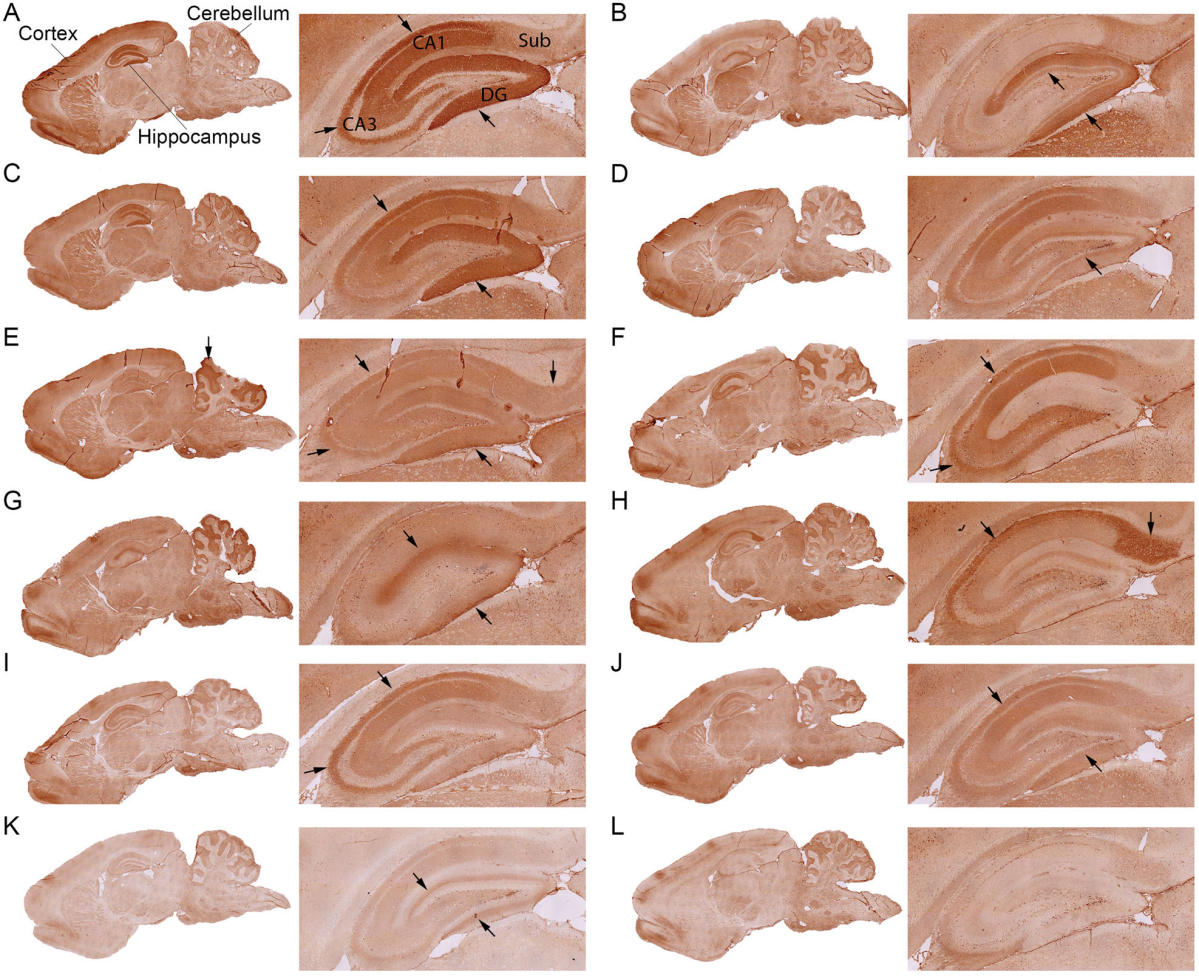
Novel rat brain immunohistochemistry patterns of autoantibodies from NESDA cohort individuals. **A**–**K** represent 11 unique IHC staining patterns of plasma autoantibodies on sagittal sections of rat brain. For each pattern, also a larger magnification of the hippocampus is shown. Arrows indicate regions with the strongest immunoreactivity in the hippocampus or cerebellum. The CA1, CA3, dentate gyrus (DG), and subiculum (Sub) regions of the hippocampus are labeled in A. **l** represents a negative staining result from a healthy individual. Scale bar = 500 µm.

Plasma samples from 2231 individuals were initially tested by IHC on rat brain tissue, as described^27^. Overall, 106 (4.8%) samples had scores of 1 or higher, of which 56 (2.4%) samples had score 1 (borderline), 42 (1.8%) had score 2 (weak positive), and 8 (0.4%) had score 3 (strong positive) (Table 2). Within the positive samples, 11 novel staining patterns were identified; they were clearly different from reported patterns of known NSAbs associated with autoimmune encephalitis^12,31^ (Fig. 1, pattern **A**–**K**; and Supplementary Fig. 2).

**Table 2:** Prevalence of neuronal autoantibodies in the 2231 NESDA respondents.

	Current depression or anxiety (*N* = 819)^a^	Depression or anxiety in remission (*N* = 920)^b^	Control group (*N* = 492)	*P* value
IHC score 1, 2, or 3^c^	44 (5.4%)	37 (4.0%)	25 (5.1%)	N/S^d^
Borderline (Score 1)	23 (2.8%)	19 (2.1%)	14 (2.8%)	N/S
Weak positive (Score 2)	15 (1.8%)	16 (1.7%)	11 (2.2%)	N/S
Strong positive (Score 3)	6 (0.7%)	2 (0.2%)	0	N/S (0.071)
Positive by IHC and staining on live neurons (Novel NSAbs)^e^	7 (0.9%)	0	1 (0.2%)	0.006
Positive by IHC and fixed CBA^f^	1 (0.1%)	1 (0.1%)	0	
Anti-CASPR2	0	1 (0.1%)	0	N/S
Anti-GAD65/67	1 (0.1%)	0	0	N/S
Others^g^	0	0	0	N/S
Positive by IHC and live CBA^f^	3 (0.4%)	8 (0.9%)	2 (0.4%)	
Anti-NMDAR/Anti-AMPAR	0	0	0	N/S
Anti-CASPR2	1 (0.1%)	5 (0.5%)	2 (0.4%)	N/S
Anti-LGI1	0	1 (0.1%)	0	N/S
Anti-GABAAR	0	1 (0.1%)	0	N/S
Anti-GABABR	2 (0.2%)	1 (0.1%)	0	N/S

^a^Depression and/or anxiety present in the 6 months prior to assessment.

^b^Lifetime depression and/or anxiety diagnosis, but not in the 5 months prior to assessment.

^c^In all, 2231 were tested by IHC.

^d^N/S: no significant difference. *α* = 0.05

^e,f^Samples with IHC score 1, 2, or 3 (*N* = 106) were further tested by staining on live neurons and CBA.

^g^Anti-NMDAR, anti-AMPAR, anti-LGI1, anti-GABAAR, and anti-GABABR.

From the current depression or anxiety group, 44 out of 819 samples (5.3%) had scores of 1 or higher. From the depression or anxiety in remission group, 37 out of 920 samples (4.0%) had scores of 1 or higher and from the control group, 25 out of 492 samples (5.1%) had scores of 1 or higher. The percentages of positive samples were not significantly different between the groups (Table 2). Interestingly, all eight samples with score 3 were found in the depression or anxiety groups (current or in remission, see Table 3, P1–8) and none in the control group (6/819 vs 2/920 vs 0/492, *P* = 0.071, Table 2).

**Table 3:** Characteristics of the individuals with autoantibodies giving score 3 by IHC or positive for known or to novel NSAbs at wave 3.

No.	Sex	Age (year)	IHC score/pattern^a^	Titer by IHC	CBA	Staining on live neurons	Diagnosis^b^	Anxiety/depression onset age (year)	Anti-depressants usage during the last 2 years	Fever/cold^c^	Chronic comorbidities
P1	Male	66	3/A	Not tested	Negative	Negative	Remitted depression	–/35	No	No	Rheumatism: fibromyalgia; hay fever, house dust allergy
P2	Female	46	3/B	12800	Negative	Positive	Current anxiety	8/–	No	Cold	Hay fever
P3	Male	55	3/B	6400	Negative	Positive	Current anxiety, remitted depression (single episode)	53/14	No	Cold	Hypertension
P4	Female	58	3/B	800	Negative	Positive	Current anxiety and depression	56/56	No	Cold	Chronic bronchitis; diarrhea; eczema; psoriasis
P5	Female	57	3/C	3200	Negative	Positive	Current anxiety	55/–	No	Cold	Hypertension; breast cancer; eczema; arthritis; renal pelvic inflammation with encapsulated kidney stone; chronic heart conditions (unspecified)
P6	Female	31	3/C	3200	Negative	Positive	Current anxiety, remitted depression (single episode)	29/20	No	No	No
P7	Female	58	3/C	Not tested	Negative	Negative	Remitted anxiety and depression	16/36	No	No	Diabetes; arthritis; benign connective tissue tumor; allergy
P8	Male	66	3/D	Not tested	Negative	Negative	Current depression	–/45	Yes	No	No
P9	Male	56	2/E	1600	Negative	Positive	Current anxiety, remitted depression (single episode)	48/28	No	No	Eczema
P10	Female	36	2/F	1600	Negative	Positive	Current anxiety and depression	35/34	No	No	Chronic heart conditions (unspecified); ligament injury
P11	Male	53	1/H	Not tested	CASPR2 positive (live and fixed CBA)	Negative	Remitted depression	–/51	Yes	No	Hypertension; asthma; arthritis; hay fever;
P12	Male	66	2/GAD pattern	Not tested	GAD65/67 positive	Negative	Current depression	–/61	No	No	Diabetes, hypertension; arteriosclerosis, heart disease, chronic bronchitis; rheumatism; arthritis; constipation;
C1	Male	20	2/K	400	Negative	Positive	None (Control group)	–/–	No	No	Injury (overloading of the knee)

^a^IHC novel pattern: 11 different novel IHC patterns were found in this study (see Fig. 1, **A**–**K**).

^b^Current: diagnosed with depression and/or anxiety within 6-month when blood samples were collected. Remitted: diagnoses with depression and/or anxiety earlier in life but no diagnoses of depression or anxiety within 6-month when blood samples were collected.

^c^Had a fever or a cold in the past week before the blood sample was drawn based on questionnaires.

### Staining on live neurons: seven out of eight individuals with unknown NSAbs had current anxiety or depression

**Fig. 2:**
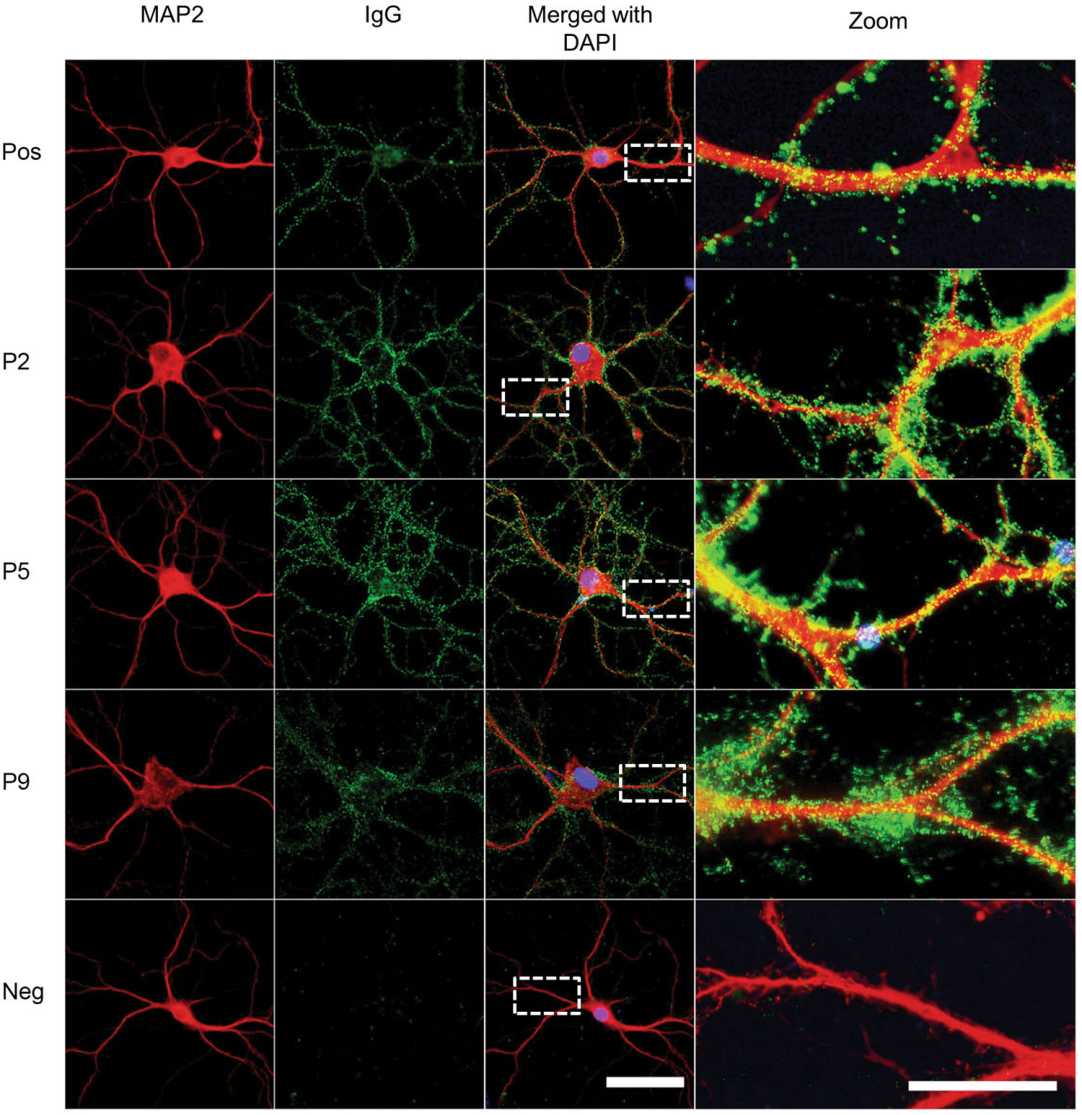
Immunofluorescence staining on primary hippocampal live neurons of plasma samples from selected individuals. Neurons were stained with anti-MAP2 (red), autoantibodies were stained with anti-human IgG (green) and nuclei were stained with DAPI (blue). Serum from an encephalitis patient with NMDAR autoantibodies was used as a positive control (“Pos”), plasma from a healthy individual was used a negative control (“Neg”). Plasma samples from Case 2, Case 5, and Case 9 showed clear immunoreactivity to live neurons. Scale bar = 50 µm. Regions in white boxes are shown at higher magnification on the right panels; these show a speckled human IgG staining along the dendrite. Scale bar = 20 µm.

Autoantibodies to neuronal membrane proteins are much more likely to be pathogenic than autoantibodies to intracellular antigens. As both could bind to brain tissue by IHC, we tested all the 106 samples that had IHC scores of 1 or higher for NSAbs on live-cultured neurons at a dilution of 1:50 (see Table 2). Overall, NSAbs were present in 8 out of 106 samples. From the current depression or anxiety group, 7 out of 44 samples were positive (P2–6, P9, and P10, see Table 3; three of the strongest stainings are shown in Fig. 2). All seven had anxiety, two of them also had depression. In the depression or anxiety in remission group, none of the 37 samples had NSAbs. From the control group 1 out of 25 samples was positive (C1, see Table 3). These eight samples accounted for 0.4% of the whole cohort. The prevalences of NSAbs in each of the groups (current depression or anxiety group vs depression or anxiety in remission group vs controls) differed significantly (7/819 vs 0/920 vs 1/492, *P* = 0.006, Fisher’s exact test). The NSAbs were found more frequently in the current depression or anxiety group compared with the group in remission (7/819 vs 0/920, *P* = 0.015, with Bonferroni correction of multiple comparisons), whereas there was no significant difference between the current depression or anxiety group and the controls (7/819 vs 1/492, *P* = 0.27), nor between the group in remission and the controls (0/920 vs 1/492, *P* = 0.35).

### CBA studies: autoantibodies to known neuronal antigens were rarely present

To test if plasma samples contained autoantibodies to known neuronal antigens, all 106 samples with IHC scores of 1 or higher were screened for NSAbs using both fixed and live CBAs, and tested for GAD65 and GAD67 antibodies by fixed CBA (Table 2). By fixed CBAs, two samples were found positive. One sample (P11, see Table 3) was positive for anti-CASPR2 and the other one (P12, see Table 3) was positive for both anti-GAD65 and anti-GAD67. P12 had the corresponding IHC pattern (image not shown), but this was not the case for P11 (Supplementary Fig. 3). By live CBA, 13 samples were found positive: 8 for CASPR2 (including P11), 1 for LGI1, 1 for GABAAR, and 3 for GABABR. All were very weakly positive (titration between 1:20 to 1:250) compared to the positive control samples from encephalitis patients we used. None of these live CBA-positive samples had tested positive for NSAbs on live neurons, or presented a correlating antigen-binding pattern on rat brain IHC. In addition, a pre-absorption assay was performed for all CASPR2-positive samples identified by live CBA. Plasma was first pre-absorbed on (human) CASPR2 transfected cells and subsequently incubated on rat brain (Supplementary Fig. 3). However, the staining intensity in IHC was not reduced, indicating that the immunoreactivity to the hippocampus was not caused by autoantibodies binding to human CASPR2.

Except for P11, the very weak positive cases found by live CBAs did not correlate with any other tests and therefore the live CBA results were considered inconclusive. To summarize, the studies detected one patient with confirmed CASPR2 and one with confirmed GAD antibodies.

### Cross-validation of autoantibody detection

The 18 samples that gave the strongest staining intensity by IHC (the eight samples described above as positive on live neurons were all included) and two negative samples were retested in a reference diagnostic laboratory according to established diagnostic approach for autoimmune encephalitis (the test conditions and strategy are described in the supplementary methods). By IHC on rat brain, the same staining patterns and intensities were reproduced for all samples (Supplementary Fig. 4). Staining patterns in 11 samples were identified as potentially relevant for NSAbs and these samples were therefore further tested on live neurons with a sample dilution of 1:200. These 11 selected samples included seven out of the eight that had been previously identified to contain novel NSAbs (P10 was not included). The presence of NSAbs to cultured neurons was confirmed for three individuals (P2, P3, and P9, Supplementary Fig. 4).

Based on the observed IHC staining patterns, seven samples were then also tested by fixed CBA for autoantibodies to NMDAR, LGI1or GAD65/67 at a 1:200 dilution in each case, but none of the samples tested positive (Supplementary Fig. 4).

### Analysis of immunoreactivity of NSAbs and clinical correlations

We aimed to characterize the seven individuals with NSAbs and coexisting anxiety or depression by further IHC tests and by analyzing their clinical information (see Table 3). All these patients were diagnosed with current anxiety, whereas the anxiety sub-diagnoses were diverse (three had agoraphobia, two had generalized anxiety disorders, one had social phobia, and one had panic without agoraphobia). The BAI (Beck Anxiety Inventory) and the IDS (Inventory for Depressive Symptomatology) were not different compared with other patients with current anxiety or depression (data not shown). None of them had used anti-depressant drugs in the 2 years before blood sampling. One patient had breast cancer, had undergone an operation and received chemotherapy. The control individual that had tested positive for NSAbs (C1) had a history of injury (overloading of the knee) without other systemic health conditions.

Individuals with NSAbs and current anxiety or depression had IHC staining patterns B, C, E, and G, whereas pattern K was only found in the control individual with NSAbs.

Because not only the presence but also a high concentration of NSAbs can be an indicator of autoimmune brain disease^32^, we titrated plasma samples by brain IHC, as this method is more sensitive compared with immunofluorescent staining on live neurons for unidentified autoantibodies based on our experience. The titers of the seven patients with current anxiety and NSAbs were equal or above 1:800, whereas the control sample had a titer of 1:400.

As depression and anxiety are often chronic conditions, we tested additional plasma samples that were donated 2 years earlier (“Wave 1”) and 4-year later (“Wave 5”) by the individuals that had NSAbs at Wave 3. We noted no obvious correlation between changes of antibody titer by IHC and the presence of disease (anxiety and/or depression). In one individual (P2, see Supplementary Fig. 5) the NSAb and IHC reactivity was not found in earlier or later plasma samples, which coincided with the absence of anxiety and depression at these time points. In the remaining six individuals with anxiety or depression, the IHC staining patterns were stable over time in all available plasma samples (Supplementary Fig. 5, P3–6, P9, and P10). In one control individual (C1), NSAbs were found in the plasma of Wave 1 and Wave 3, but not of Wave 5.

## Discussion

To our knowledge, this is the largest study so far to investigate whether neuronal autoantibodies, especially NSAbs, are associated with depression or anxiety. Our results indicate that these autoantibodies are not present in the vast majority of individuals with these disorders, which aligns with findings from earlier reports^23,25,33^. Nevertheless, none of the previous studies investigated the presence of potentially novel NSAbs. Our data show that a small fraction (0.9%) of patients with current anxiety or depression had autoantibodies targeting yet undefined surface antigens, compared with individuals with depression or anxiety in remission where no such autoantibodies were found.

We need to interpret these findings carefully and to consider their significance because novel NSAbs could be innocent bystanders unrelated to the disorder. In this context, false positives could also pose a considerable problem for the interpretation of laboratory data, especially for diseases with low prevalence such as NSAbs-associated disorders^33,34^. Weak positive samples were found in all groups by IHC or live CBA, which could not be confirmed by a second method. These autoantibodies were not related to depression or anxiety but may be a result of unrelated autoimmune conditions since individuals with somatic diseases were not excluded from the groups.

The use of multiple techniques in tandem, e.g., IHC followed by neuronal staining, is commonly used to increase the specificity of antibody detection for the diagnosis of autoimmune encephalitis^26^. When analyzing the anxiety and depression cohort, only seven samples fulfill the criteria to be both positive by IHC and also by staining on live neurons. These samples were comprehensively screened by CBAs, and so far, the targeted surface antigens remain unknown. Notably, all were from current anxiety patients (with or without depression), but none was from a patient in remission. However, in the present study, one sample (0.2%) with NSAbs was also found in the control group, which questions the clinical relevance of those novel NSAbs found in the depression or anxiety groups. Our explanation is that the NSAbs present in the depression or anxiety groups targeted different neuronal surface proteins from the one found in the control group because they gave obviously different staining patterns on brain tissue. Interestingly, two patterns (patterns B and C) were repeatedly found in patients with depression and anxiety disorders. These patterns were absent in our previous study where we used a similar strategy to detect NSAbs in individuals with psychotic disorders (*n* = 621) and control participants (*n* = 257 blood donors)^27^, which supports the notion of genuine novel NSAbs in the current study.

In a reference laboratory, the presence of novel NSAbs was confirmed in three samples, when analyzed according to the procedure and evaluation criteria followed routinely for diagnosing autoimmune encephalitis. If we considered only these three sera, our study lacked the power to detect significant differences between the tested groups. Some IHC staining patterns we identified were considered unspecific for NSAbs associated with autoimmune encephalitis. Moreover, the test of NSAbs in cultured neurons used a higher sample dilution in the reference laboratory, compared to the one we used. These differences highlight the importance of confirmational studies, the standardization of methods and interpretations, especially when using patient cohorts with different characteristics/symptoms.

This cohort study has several limitations: (1) not all the known NSAbs that have been reported to be associated with neurological diseases were tested by CBA in this study [for instance, not tested were antibodies to D2DR, mGluR5, mGluR1, neurexin-3α, IgLON5, DNER (Tr), Glycine receptor and amphiphysin]. But the presence of high levels of such antibodies was excluded empirically based on the IHC patterns. (2) As we chose the strategy to screen all plasma samples first by IHC, we cannot exclude the possibility that we missed samples with low titer or low-affinity NSAbs that can only be detected by CBA. (3) As mentioned, we only analyzed peripheral blood samples, whereas no cerebrospinal fluid was available, so the question as to whether identified NSAbs would pass the blood–brain–barrier remains to be investigated.

To conclude, we suggest that further studies aiming to validate these findings should focus on individuals currently suffering from anxiety (with or without depression) by using cerebrospinal fluid and blood samples. Considering the low prevalence of the novel NSAbs found in this study, identification of the autoantigens, e.g., by mass spectrometry or a longitudinal study by timely follow-up would help to further reveal the possible clinical relevance.

## Supplementary information

Supplementary methods

Supplementary Table 1

Supplementary Figure legends

Supplementary Figure 1

Supplementary Figure 2

Supplementary Figure 3

Supplementary Figure 4

Supplementary Figure 5
