# Supplementary material for: Novel neuronal surface autoantibodies in plasma of patients with depression and anxiety
Source: Transl Psychiatry. 2020 Nov 23;10:404. doi: 10.1038/s41398-020-01083-y (PMC7683539; doi:10.1038/s41398-020-01083-y)
Supplement: Supplementary file 1 — Supplementary methods [file 41398_2020_1083_MOESM1_ESM.docx]

**Supplementary Methods**

### **Source of plasmids**

Clones containing full-length human cDNA sequences coding for the GRIN1 (NM_000837.1) and GRIN2B (NM_000834.4) receptor were obtained from the Thermofisher EST collection (Thermofischer Scientific). GRIN1 was digested with Psil and GRIN2B was digested with EcoRI, and were cloned into pcDNA 3.1 digested with EcoRV and transformed in DH10B cells (NEB, C4040-03). Both plasmids were sequenced (GATC Biotech) and confirmed to correspond with the GRIN1 and GRIN2B reference sequences. For the live CBA, we used human GRIN1 in pIRES-eGFP^1^ that was kindly provided by Fabienne Brilot-Turville (University of Sydney). Human AMPAr was expressed from human GluR1 (pTriEx1backbone) and GluR2 (pDest-40 backbone) and human GABABR from GABBR1(pDest-40 backbone) and GABBR2 (pTriEx1backbone). Human LGI-1 was cloned in frame with the transmembrane region of CASPR2 into pcDNA3 to generate membrane-bound LGI-1 and with mCherry for the live CBA (pIRES2-DsRed2 backbone). The cloning details were described previously ^2^. These 6 plasmids were a kind gift from Patrick Waters (University of Oxford). GABAAR plasmids were obtained from Erdem Tuzun (Istanbul University, Turkey)^3^ and expressed the human alpha1, beta2, and gamma1 subunit. Human CASPR2 was received from Catherine Faivre-Sarrailh (CNRS, Marseille)^4^ with a pcDNA3 backbone with a mCherry ^5^ tag for the live CBA and without the tag for fixed CBA. The GAD plasmids expressed human GAD65 and GAD67 from the pCMV6-XL5 plasmid which was a kind gift from Francesc Graus (IDIBAPS, Barcelona) ^6^.

**Serological analyses**

**Immunohistochemistry (IHC) grading:** After the staining, slides were scanned by VENTANA iScan HT scanner at 20 times resolution. The images generated were scored from 0 to 3 and “inconclusive” by two experienced observers using Ventana Image Viewer (Vision 3.1.4) according to patterns and staining density on the hippocampus (nuclei staining was not taken into account). Generally, when there was an absence of staining in the hippocampus as in non-disease controls, it was considered negative and scored 0. When a clear pattern observed, it was considered positive and a score was given. Positive samples were scored from 1 to 3 depending on the intensity of the staining, from less to more intense. If the staining was blurry or quality was otherwise impaired, it would be considered ‘inconclusive’; Plasma considered ≥ 1 or inconclusive at the first round of staining was repeated to validate the results and evaluated by 2 independent experienced observers. If two rounds of staining for a certain sample resulted in the same score, this was considered the final score. If the scores were different between experiments, the staining was repeated at least once more and a final score was given according to all the pictures available for this sample. Those with score 0 were considered negative, 1 was considered borderline, 2 was considered weak positive and 3 was considered strong positive.

Autoantibodies titers for IHC positive samples were tested using dilutions with a factor 2 (from 1:200 to 1:25,600) on rat brain IHC. The limit dilution factor was established as the lowest dilution which presented reactivity in the assay. For example, when a sample was positive at the dilution of 1:3,200 but becomes negative at 1:6,400, then the autoantibody titer of this sample is defined as 3,200. A similar approach was used for the CBA and the staining on live cultured neurons.

**Fixed cell-based assay (CBA):** The screening always included a positive control from a patient with autoantibodies, except for the anti-GABAAR staining for which no positive control was available. Cover glasses were mounted onto 7µl DAPI mounting medium (Vector laboratories, #H-1200) and evaluated by two independent trained observers on a BX51 Olympus microscope.

**Validation of antibody detection:** The validation of our results was performed in Barcelona. Samples were first tested by IHC and if positive, they would be tested by staining on live neurons. Certain cell-based assays for specific antigens were selected to test according to the staining pattern on rat brain tissue by IHC. For the staining on live neurons, they used a dilution of 1 in 200 compared to a dilution 1 in 50 as was done in our laboratory.

**References:**

1. Pathmanandavel K, Starling J, Merheb V, et al. Antibodies to surface dopamine-2 receptor and N-methyl-D-aspartate receptor in the first episode of acute psychosis in children. *Biol Psychiatry.* 2015;77(6):537-547.

2. Irani SR, Alexander S, Waters P, et al. Antibodies to Kv1 potassium channel-complex proteins leucine-rich, glioma inactivated 1 protein and contactin-associated protein-2 in limbic encephalitis, Morvan's syndrome and acquired neuromyotonia. *Brain.* 2010;133(9):2734-2748.

3. Pettingill P, Kramer HB, Coebergh JA, et al. Antibodies to GABAA receptor alpha1 and gamma2 subunits: clinical and serologic characterization. *Neurology.* 2015;84(12):1233-1241.

4. Bonnon C, Goutebroze L, Denisenko-Nehrbass N, Girault JA, Faivre-Sarrailh C. The paranodal complex of F3/contactin and caspr/paranodin traffics to the cell surface via a non-conventional pathway. *J Biol Chem.* 2003;278(48):48339-48347.

5. Pinatel D, Hivert B, Boucraut J, et al. Inhibitory axons are targeted in hippocampal cell culture by anti-Caspr2 autoantibodies associated with limbic encephalitis. *Front Cell Neurosci.* 2015;9:265.

6. Arino H, Gresa-Arribas N, Blanco Y, et al. Cerebellar ataxia and glutamic acid decarboxylase antibodies: immunologic profile and long-term effect of immunotherapy. *JAMA Neurol.* 2014;71(8):1009-1016.
