# Supplementary material for: Novel neuronal surface autoantibodies in plasma of patients with depression and anxiety
Source: Transl Psychiatry. 2020 Nov 23;10:404. doi: 10.1038/s41398-020-01083-y (PMC7683539; doi:10.1038/s41398-020-01083-y)
Supplement: Supplementary file 2 — Supplementary Table 1 [file 41398_2020_1083_MOESM2_ESM.docx]

**Supplementary Table 1: Antibodies used for cell-based assay**

| **Antibody** | **source** | **dilution** |
| --- | --- | --- |
| Anti-GluN1 | #PAB12310, Abnova | 1：500 |
| Anti-LGI1 | #AB30868, Abcam | 1：1000 |
| Anti-CASPR2 | # AB33994, Abcam | 1：1000 |
| Anti-GABAAR | #75136, Antibodies Incorporated | 1：20000 |
| Anti-GABABR | #sc14006, Santa Cruz Biotechnology | 1：500 |
| Anti-GAD65 | 7309LB, Christina Hampe, (University of Washington) | 1：1000 |
| Anti-GAD67 | 10266/20B, Christina Hampe, (University of Washington) | 1：1000 |
|  |  |  |
| Goat-anti-human-IgG Fcγ-Alexa488^1^ | #109-546-170, Jackson | 1：1000 |
| Goat-anti-rabbit-Alexa594 | #111-585-144, Jackson | 1：1000 |
| Goat-anti-mouse-Alexa594^2^ | #A11005, Invitrogen | 1：1000 |

1. The dilution for live CBA is 1: 750.

2. Used only for Anti-GABAAR detection
