# Supplementary material for: Novel neuronal surface autoantibodies in plasma of patients with depression and anxiety
Source: Transl Psychiatry. 2020 Nov 23;10:404. doi: 10.1038/s41398-020-01083-y (PMC7683539; doi:10.1038/s41398-020-01083-y)
Supplement: Supplementary file 3 — Supplementary Figure legends [file 41398_2020_1083_MOESM3_ESM.docx]

**Supplementary Figure legends**

**Supplementary Figure 1. Working flow chart and examples of positive results of neuronal surface autoantibody detection. A)** Scheme of groups of the NESDA cohort (wave 3, n=2231). Groups were defined based on whether anxiety or depression was currently present or in remission. **B)** Plasma samples from all participants were tested for the presence of neuronal autoantibodies firstly by immunohistochemistry (IHC) on rat brain**. C)** IHC positive samples were then tested on live-cultured hippocampal neurons, and **(D)** by cell-based assay (CBA) to 8 known neuronal antigens (*Pre-absorption tests were performed when samples were positive for CASPR2 autoantibodies by live CBA). The images show examples of positive results for each method. **B)** IHC staining of a sagittal brain section given by an NMDAR autoantibody-positive sample from an encephalitis patient; the image on the right shows details of the specific hippocampus staining pattern, scale bar = 500 μm. **C)** Images show a neuronal marker MAP2 (red) and strong neuronal IgG staining from the serum of an encephalitis patient with DPPX autoantibodies (green). **D)** Images show transfected HEK cells with human GABABR (red), IgG staining from the serum of an encephalitis patient with GABABR autoantibodies (green). For C and D, the nuclei are stained with DAPI (blue), scale bar = 50 μm.

**Supplementary Figure 2.** **IHC results grouped by staining patterns.** In total, 50 samples were positive by IHC. 20 samples are not shown here because they could not be unequivocally attributed to these patterns. P1-P11 and P13-P22 were samples from individuals with depression or anxiety disorders (P12 was not shown here because it was with a known pattern (Anti-GAD65/67). C1-C9 were samples from control individuals. The images with green frames indicate the corresponding samples were also tested positive by staining on live neurons. Scale bar = 500 µm.

**Supplementary Figure 3. CBA and IHC analysis of plasma samples before and after depletion of autoantibodies on CASPR2 transfected HEK cells.** A) live CBA for CASPR2, before and after pre-absorption, showing CASPR2-mCherry (red), human IgG (green) and nuclei (blue). B) Rat brain sections were used for IHC staining, human IgG was labeled with DAB (brown). “Pos’’ corresponds to a positive serum control from an encephalitis patient with CASPR2 autoantibodies, which staining intensity was negligible after pre-absorption. “P11” is a plasma sample from the NESDA cohort which was weakly positive for CASPR2 by fixed and live CBA (A: P11 before absorption, the green signal was enhanced in this image to show the IgG reaction on transfected cells) but its IHC pattern was not affected by the pre-absorption. Plasma from a healthy individual was used as a negative control (“Neg”). CBA Scale bar = 20 µm. IHC Scale bar = 500 µm.

**Supplementary Figure 4. Comparison of the testing results between different laboratories of 18 samples selected.** All the samples were initially tested positive by our in housing IHC at Maastricht University (UM lab). Samples labeled in red were considered IHC positive at the reference laboratory (Ref lab), thus were further tested for live neurons. The images with green frames indicate the corresponding samples also tested positive by staining on live neurons. * P1, P2, P4, P5 and P6 were further tested for anti-NMDAR by fixed CBA, P2, P3, P4 and C1 was tested for anti-LGI1, C1 was also further tested for anti-GAD65/67 at the Ref lab, however, they were all negative. Note that P13 was considered negative by IHC at the Ref lab. Upon our request, the P13 sample was tested on live neurons at the Ref lab and the result was positive. Since this was not the standard procedure in the Ref lab, we did not include this in the final result of the validation part.

**Supplementary Figure 5.** **Antibody titers tested by IHC changing over time in NSAbs positive individuals**. Samples from different time points [wave 1 (baseline), wave 3 (2-year follow-up) and wave 5 (6-year follow-up)] were tested by IHC on rat brain tissue, using a serial dilution ranging from 1:200 to 1:12800. The presence or absence of anxiety or depression at the 3 times is indicated by a plus or minus symbol for each individual.
