# Supplementary figures and images for: Novel neuronal surface autoantibodies in plasma of patients with depression and anxiety
Source: Transl Psychiatry. 2020 Nov 23;10:404. doi: 10.1038/s41398-020-01083-y (PMC7683539; doi:10.1038/s41398-020-01083-y)

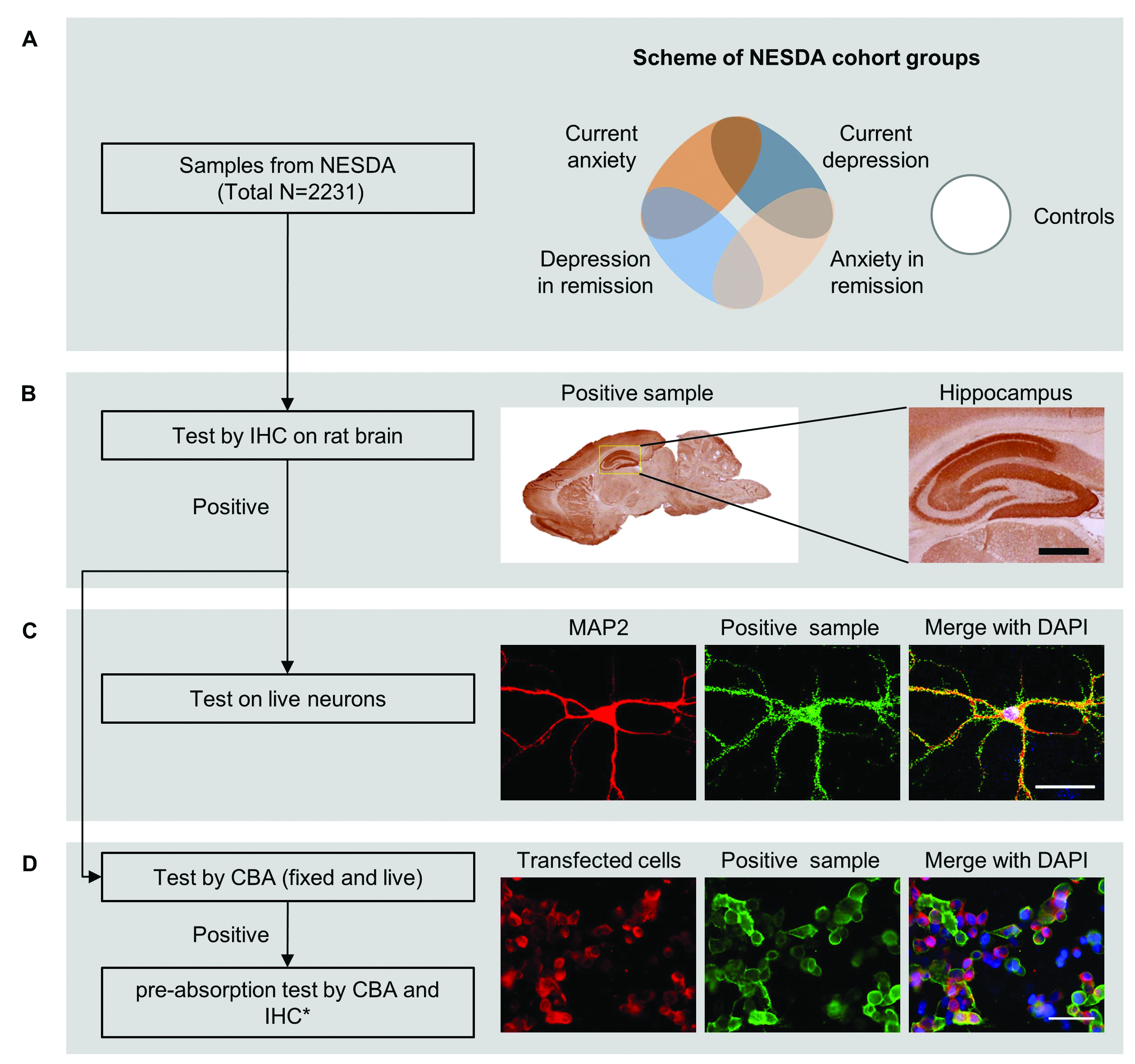

Supplement: Supplementary file 4 — Supplementary Figure 1 [file 41398_2020_1083_MOESM4_ESM.jpg]

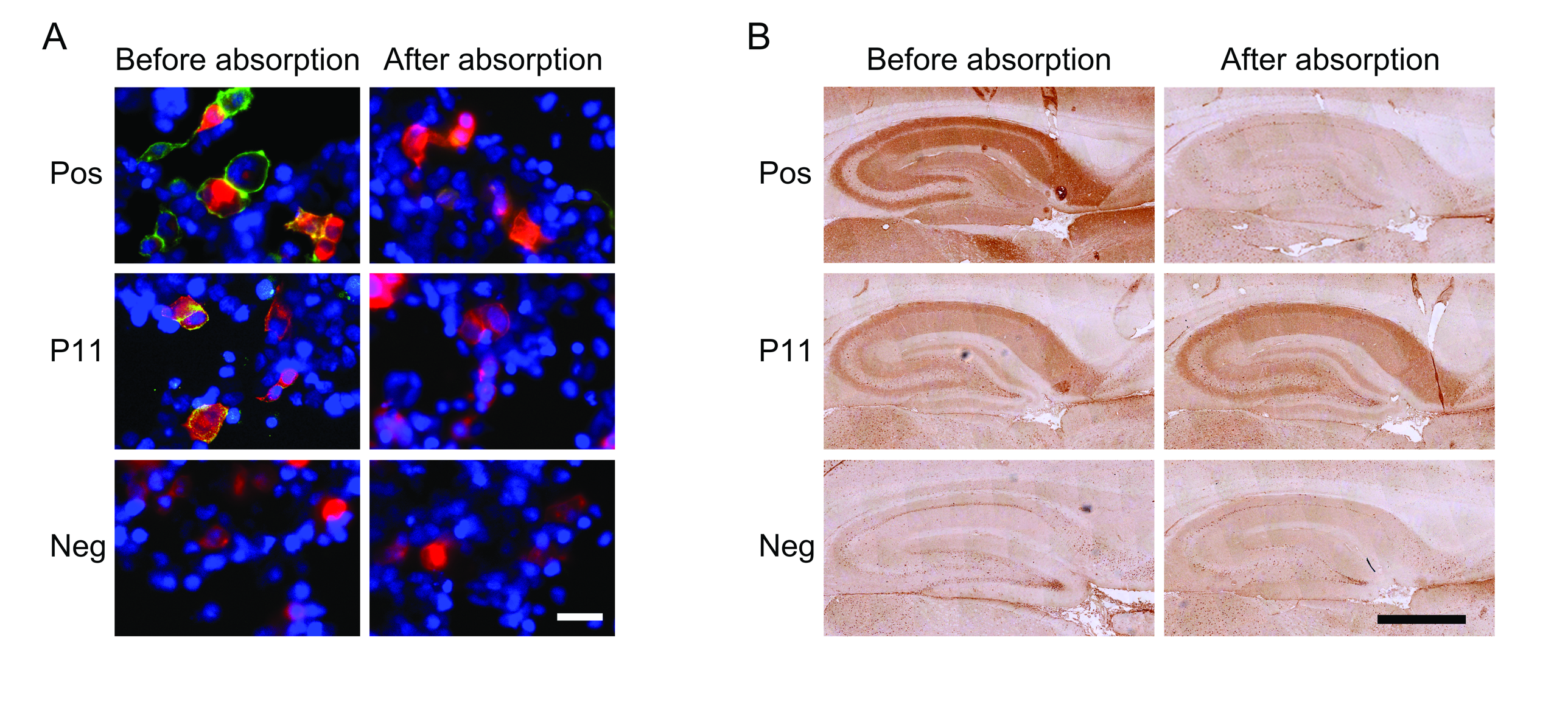

Supplement: Supplementary file 6 — Supplementary Figure 3 [file 41398_2020_1083_MOESM6_ESM.jpg]

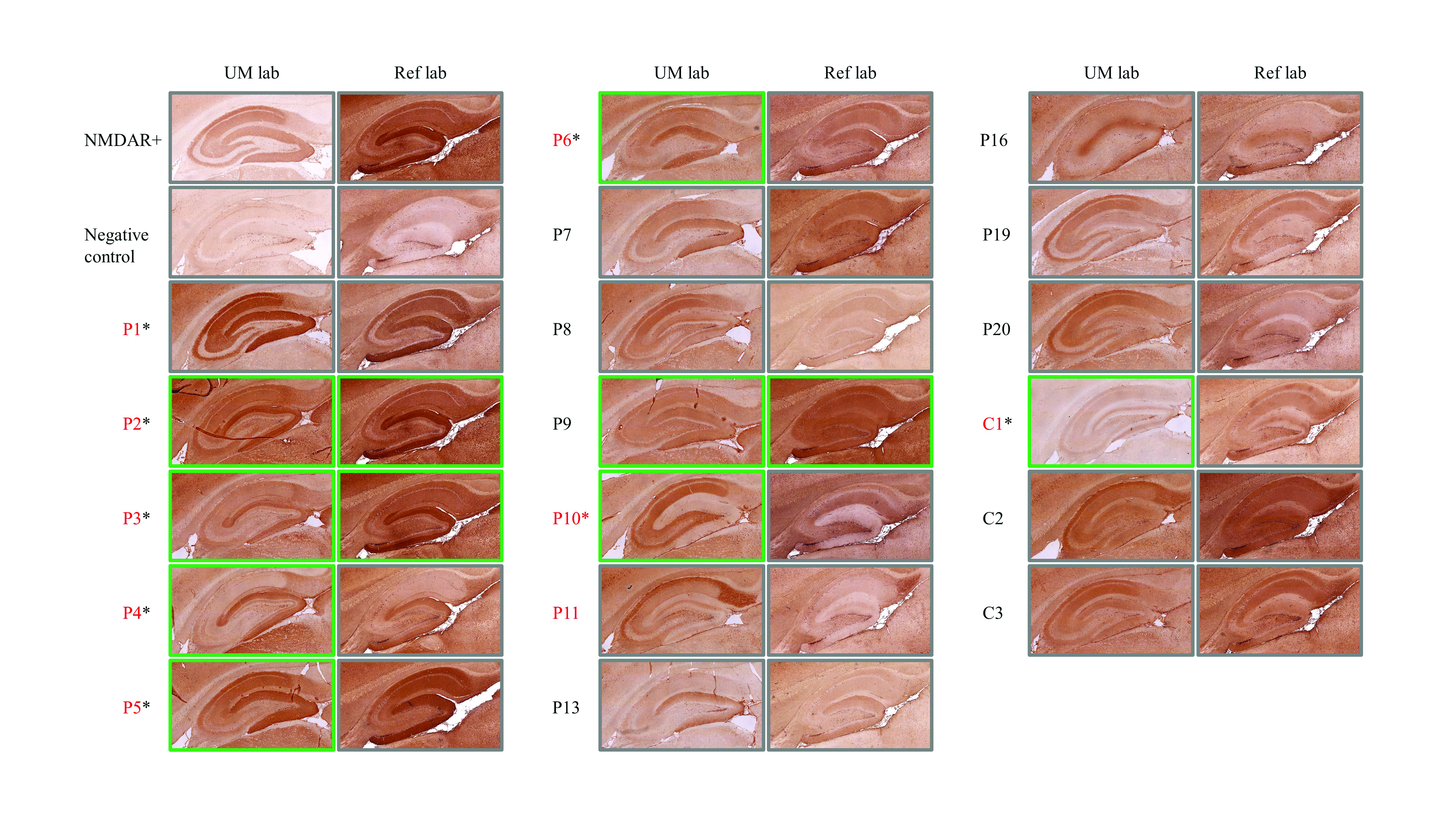

Supplement: Supplementary file 7 — Supplementary Figure 4 [file 41398_2020_1083_MOESM7_ESM.jpg]

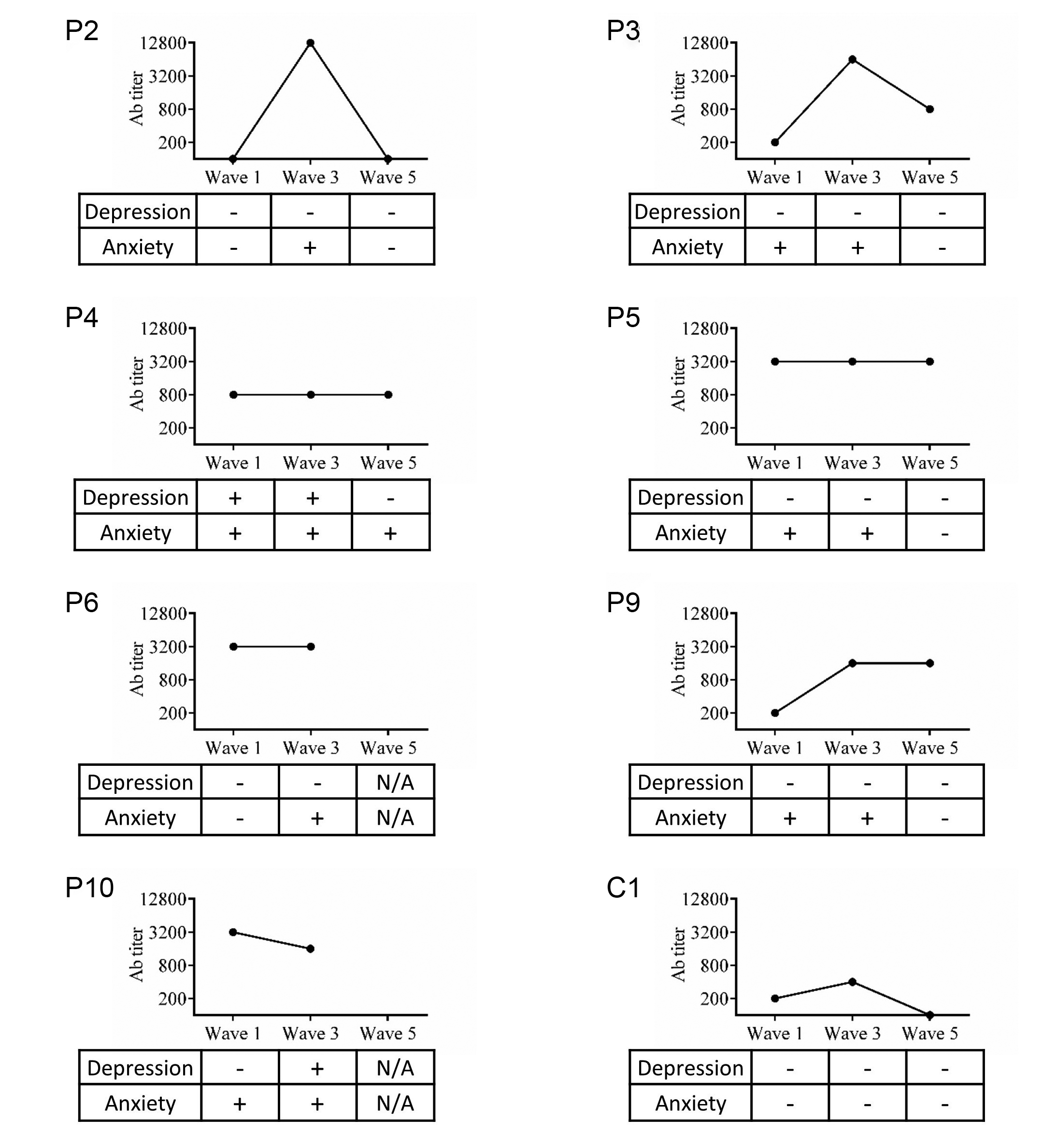

Supplement: Supplementary file 8 — Supplementary Figure 5 [file 41398_2020_1083_MOESM8_ESM.jpg]
